# The first genomic characterization of a stable, hemin-dependent small colony variant strain of *Staphylococcus epidermidis* isolated from a prosthetic-joint infection

**DOI:** 10.3389/fmicb.2023.1289844

**Published:** 2023-10-19

**Authors:** Agnieszka Bogut, Piotr Koper, Małgorzata Marczak, Paulina Całka

**Affiliations:** ^1^Chair and Department of Medical Microbiology, Medical University of Lublin, Lublin, Poland; ^2^Department of Genetics and Microbiology, Institute of Biological Sciences, Maria Curie-Skłodowska University, Lublin, Poland; ^3^Chair and Department of Forensic Medicine, Medical University of Lublin, Lublin, Poland

**Keywords:** small-colony variant (SCV), *Staphylococcus epidemidis*, prosthetic joint infection (PJI), auxotrophy, arginine deiminase pathway (ADI), Staphylococcal Cassette Chromosome, homopolymeric tracts, phase variation (PV)

## Abstract

Phenotype switching from a wild type (WT) to a slow-growing subpopulation, referred to as small colony variants (SCVs), supports an infectious lifestyle of *Staphylococcus epidermidis*, the leading cause of medical device-related infections. Specific mechanisms underlying formation of SCVs and involved in the shaping of their pathogenic potential are of particular interest for stable strains as they have been only rarely cultured from clinical specimens. As the SCV phenotype stability implies the existence of genetic changes, the whole genome sequence of a stable, hemin-dependent *S. epidermidis* SCV strain (named 49SCV) involved in a late prosthetic joint infection was analyzed. The strain was isolated in a monoculture without a corresponding WT clone, therefore, its genome was compared against five reference *S. epidermidis* strains (ATCC12228, ATCC14990, NBRC113846, O47, and RP62A), both at the level of the genome structure and coding sequences. According to the Multilocus Sequence Typing analysis, the 49SCV strain represented the sequence type 2 (ST2) regarded as the most prominent infection-causing lineage with a worldwide dissemination. Genomic features unique to 49SCV included the absence of the Staphylococcal Cassette Chromosome (SCC), ~12 kb deletion with the loss of genes involved in the arginine deiminase pathway, and frameshift-generating mutations within the poly(A) and poly(T) homopolymeric tracts. Indels were identified in loci associated with adherence, metabolism, stress response, virulence, and cell wall synthesis. Of note, deletion in the poly(A) of the *hemA* gene has been considered a possible trigger factor for the phenotype transition and hemin auxotrophy in the strain. To our knowledge, the study represents the first genomic characterization of a clinical, stable and hemin-dependent *S. epidermidis* SCV strain. We propose that previously unreported indels in the homopolymeric tracts can constitute a background of the SCV phenotype due to a resulting truncation of the corresponding proteins and their possible biological dysfunction. Streamline of genetic content evidenced by the loss of the SCC and a large genomic deletion can represent a possible strategy associated both with the SCV phenotype and its adaptation to chronicity.

## Introduction

*Staphylococcus epidermidis* (SE), a common member of the human skin microbiota, has emerged as an important causative agent of infections related to implanted medical devices (Post et al., 2017; Both et al., 2021; Fernández-Rodríguez et al., 2021; Liu et al., 2021). Infectious lifestyle of this microorganism is supported by phenotype switching from a wild type (WT) to a slow-growing subpopulation characterized by distinctive pathogenic traits and referred to as small colony variants (SCVs) (Proctor et al., 2006; Liu et al., 2021). The study of Edwards (Edwards, 2012) demonstrated that this transition occurs via a constitutive mechanism depending upon bacterial replication. It leads to the generation of a dynamic, antibiotic-resistant subpopulation able to revert to its parental phenotype. Additionally, a variety of environmental pressures including low temperature, antibiotic and disinfectant exposure, pH and oxidative stress as well as a prolonged growth under nutrient limiting conditions and intracellular environment have been reported to promote staphylococcal SCV formation (Vesga et al., 1996; Schaaff et al., 2003; Bayston et al., 2007; Onyango et al., 2013; Vestergaard et al., 2016; Perez and Patel, 2017, 2018; Lee et al., 2020; Islam et al., 2022).

According to the definition proposed by Tuchscherr et al. (2020), SCVs represent bacterial subpopulation formed within host cells, characterized by a variable phenotypic stability, slow growth rate, decreased expression of virulence factors and multifaceted alterations in metabolic pathways and/or global regulatory genes. Although there is no common metabolic change typical for SCVs, many clinical and laboratory-derived variants can be tied by a common thread which are alterations in an electron transport. These defects lead to a reduced amount of ATP available for many cellular processes including the cell wall biosynthesis, uptake of amino acids and carbohydrates; the resultant effect is a lower growth rate and production of smaller colonies (Proctor et al., 2006; Kahl et al., 2016; Proctor, 2019). Additionally, interruption of electron transport results in the accumulation of the NADH dinucleotide, which downregulates the citric acid cycle enzymes (Proctor, 2019). The decreased electron transport activity has also been related to antibiotic resistance, since an electrochemical gradient is required for the import of positively charged molecules including aminoglycosides into bacterial cells (Proctor et al., 2006). Mutations in the genes encoding for enzymes involved in the electron transport, such as those controlling menaquinone and cytochrome biosynthesis, result in the formation of respiratory-deficient SCVs which are auxotrophic for menadione and heme, respectively (Proctor, 2019; Bogut and Magryś, 2021).

There is increasing evidence that staphylococcal SCVs, including those produced by SE, can evolve from the WT clone during an infectious process (Baddour et al., 1988; Adler et al., 2003; Tuchscherr et al., 2011, 2016; Lin et al., 2016; Liu et al., 2021). Selection of SCVs is considered a bet-hedging strategy (Edwards, 2012; Liu et al., 2021) due to their higher adaptiveness compared to WTs and survival advantage associated with an ability to invade and maintain within host cells (Magryś et al., 2015; Perez and Patel, 2018).

Epigenetic, genetic and transcriptional factors are assumed to be implicated in the switch of the WT to its persistent SCV form (Bui and Kidd, 2015). Many SCVs isolated from clinical specimens can revert to a rapidly growing parental state. Non-stable (“dynamic”) SCVs are assumed to originate from regulatory mechanisms involving global regulators and non-defined mutations in response to changing environmental conditions. Intracellular milieu promotes later adaptations and formation of permanent (stable) SCVs which can carry specific mutations in their metabolic pathways (Tuchscherr et al., 2020; Liu et al., 2021).

Mechanisms underlying formation of SCVs by SE and involved in the shaping of their pathogenic potential are of particular interest for stable variants as they have been only rarely isolated from clinical samples (Liu et al., 2021). Stability, in turn, implies the existence of genetic changes. To the best of our knowledge, the genetic background of stable, clinically derived SCVs has been unveiled for only two staphylococcal strains, one represented by *Staphylococcus aureus* (SA) (Loss et al., 2019) and one strain of SE (Liu et al., 2021). Additionally, Bui and Kidd (2015) published a genomic comparison and transcriptomic analysis of a clinical SA isolate and the corresponding stable SCV which was induced experimentally by a prolonged exposure of the parental form to an inflammatory environment and steady-state growth conditions with low nutrients (Bui et al., 2015a,b).

In an attempt to investigate genetic mechanisms standing behind the SCV phenotype in the clinical context, we determined the whole genome sequence of a stable clinical SE SCV (named 49SCV) strain and compared it against five reference SE genomes. The strain was isolated from the sonicate fluid. The sample derived from a patient who had undergone a prosthetic hip joint revision surgery due to a clinical diagnosis of an aseptic implant loosening. Microbiological investigation revealed that the patient fulfilled criteria of a late prosthetic joint infection (PJI) (Parvizi et al., 2011). General characteristics of 49SCV were described previously. Briefly, the strain demonstrated hemin auxotrophy and gentamicin resistance (MIC >256 μg/mL) and was the *icaADBC* operon-positive, proficient biofilm producer under *in vitro* conditions (Bogut et al., 2014). A failure to cultivate the WT counterpart of 49SCV as well as its stability implied a furthest degree of chronicization of the infectious process leading to conversion of the original invading strain to the SCV variant and the existence of specific genetic changes involved in the phenotype transition.

This study represents the first genomic characterization of a stable and hemin-dependent SE SCV strain of clinical origin. We propose novel genetic determinants that can be involved in the phenotype switching and adaptation of SE to chronic, biofilm-associated and clinically indolent PJIs.

## Materials and methods

### Whole-genome sequencing

Genomic DNA of the 49SCV strain was extracted using the Genomic Micro AX *Staphylococcus* Gravity kit (A&A Biotechnology, Poland) according to the manufacturer’s protocol (including lysostaphin at a concentration of 4 U/μl and proteinase K at a concentration of 20 mg/mL). The purity and concentration of the DNA sample were verified using the BioTek Synergy LX spectrophotometer (Agilent Technologies, US).

The DNA sequencing was performed using the MiSeq benchtop sequencer (Illumina, San Diego, CA, USA). Sequencing of pair-end 150 nt libraries resulted in 1,708,902 reads. The reads were trimmed for quality and adaptor sequences were removed using Trimmomatic (Bolger et al., 2014). Genome assembly was carried out using the Unicycler tool (Wick et al., 2017). The genome sequence is available in the GenBank database with the accession number GCF_024505205.1.

The PGAP (Tatusova et al., 2016) tool was used to annotate the genome and the process was a part of submitting the genome to the GenBank database. Similarly, genome completeness was verified as a part of the submissions using the CheckM (Parks et al., 2015) tool.

### Phylogenetic analysis

The AutoMLST (Alanjary et al., 2019) web server was used to determine the phylogenetic position of 49SCV. In addition, individual contigs were scanned against the PubMLST typing schemes using the mlst (Seemann, 2023a) tool. The iTol was used for the tree visualization (Letunic and Bork, 2019).

### Comparative genomics

A full comparative analysis of the 49SCV genome was conducted in relation to five well-annotated SE reference genomes: ATCC 12228 (GCA_000007645.1), ATCC 14990 (GCA_006094375.1), NBRC 113846 (GCA_020181395.1), O47 (GCA_013317125.1), and RP62A (GCA_000011925.1). Briefly, the 49SCV genome contigs were reordered against the O47 reference and then compared to each other using the progressive Mauve (Darling et al., 2010) algorithm. In parallel, using the same genomes, the pangenome at the level of proteins of the analyzed strains was determined using the anvi’o (Eren et al., 2021) software package.

To determine the presence of transposons in the 49SCV genome, blastn (Zhang et al., 2000) and sequences of 11 transposons found in SE genomes (Tn551, Tn552, Tn554, Tn558, Tn559, Tn4001, Tn4003, Tn5404, Tn5406, Tn5801, and Tn6072) were used as queries. The strategy was also used to search for the insertion sequence (IS) elements, except that the ISFinder database was used (Siguier et al., 2006).

For the SNP analysis filtered, clean reads were mapped against each of the referenced genomes using Snippy (Seemann, 2023b).

### Other bioinformatics

CRISPR sequences were searched for using the CRISPRfinder tool (Grissa et al., 2007). Prophages were found using the Phigaro (Starikova et al., 2020) and checked for completeness using CheckV (Nayfach et al., 2021). Unless otherwise stated, individual tools have been used with default parameters.

## Results and discussion

### General genomic features of 49SCV

The genome of the 49SCV strain was sequenced *de novo*. The trimmed and filtered reads were assembled, which resulted in a draft genome composed of 39 contigs and an average coverage of 165X. The genome size was estimated to be 2,386,088 bp, while the N50 (the sequence length of the shortest contig at 50% of the total assembly length) and L50 (the smallest number of contigs whose total length is half of the estimated genome size) parameter values were 135,189 bp and 5, respectively. A total number of 2,297 genes were predicted in the genome (versus 2,432 and 2,359 genes for ATCC 12228 and ATCC 14990, respectively), of which 2,161 were the protein-coding genes (versus 2,278 and 2,233 genes for ATCC 12228 and ATCC 14990, respectively). The percentage of GC pairs was 32.1%, which was very similar to the analogous parameters in other publicly available SE genomes. The CheckM analysis estimated the 49SCV strain genome as highly complete (98.53%) and free of contamination, which indicates a high quality of the obtained data.

### Phylogenetic analysis

**Figure 1:**
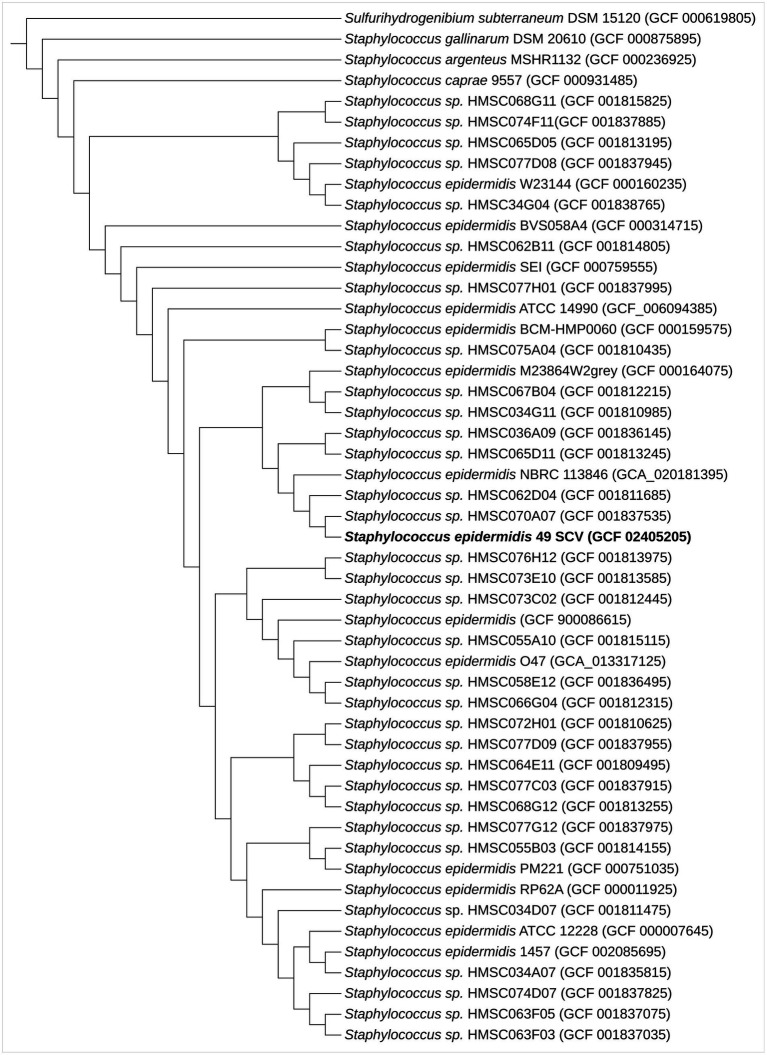
Maximum-likelihood phylogenomic tree based on 70 concatenated core genes indicating the phylogenetic positions of SE 49SCV strain with other related species. Sulfurihydrogenibium subterraneum DSM 15120 was used as an outgroup. The GenBank genome accession numbers are shown in parentheses.

The AutoMLST tool revealed that the closest related genome for 49SCV was *Staphylococcus* spp. strain HMSC070A07 (Figure 1). A search of the analyzed genome against the PubMLST database allowed to assign 49SCV to the sequence type 2 (ST2), considered as the most prominent infection-causing SE lineage with a worldwide dissemination (Miragaia et al., 2007; Du et al., 2013; Post et al., 2017; Lee et al., 2018). It has been regarded as a hospital adapted SE line, circulating within health-care facilities, being transferred from fomites or health care personnel to patients and able to subsequently cause biomaterial-associated infections (Du et al., 2013; Lee et al., 2018; Both et al., 2021). Interestingly, the catheter-sourced and stable SE SCV representing the ST2 has already been reported (Liu et al., 2021).

The ST2 typically carries pathogenesis-relevant genotypic (*icaADBC*, IS256) and phenotypic (biofilm formation) traits (Du et al., 2013; Lee et al., 2018; Both et al., 2021). Moreover, it tends to harbor a plethora of antimicrobial and antiseptic resistance genes (Lee et al., 2016, 2018; Both et al., 2021). Indeed, our previous study revealed that 49SCV was the *icaADBC*-positive, proficient biofilm producer (Bogut et al., 2014). The *ica* operon is involved in the synthesis of the polysaccharide intercellular adhesin (PIA) which contributes to the biofilm accumulation in many clinically significant SE (Fey and Olson, 2010; Rohde et al., 2010). The presence of a complete *icaADBC* cluster and a divergently transcribed regulatory *icaR* gene in the strain was further evidenced in this work (contig 22; NPW32_RS11125, NPW32_RS11120, NPW32_RS11115, NPW32_RS11110, and NPW32_RS11130, respectively). All *ica* genes showed a very high degree of similarity within the set of compared genomes. Nevertheless, the 49SCV strain did not meet the multiresistant profile suggested above. It demonstrated resistance to gentamicin in relation to its haemin-dependence (Bogut et al., 2014), a defective electron transport chain and the resultant decreased membrane potential necessary for the drug uptake.

## *Clustered regularly interspaced short palindromic repeats* (CRISPR) sequences

The presence of CRISPR sequence clusters in SE leads to a reduction in the intensity of conjugation and transformation by plasmid DNA (Marraffini and Sontheimer, 2008). The CRISPR-related genes (*cas1*, *cas2, cas6*) were not found in the genome of 49SCV, similarly to the genomes of the reference ATCC 12228 and O47 strains (Raue et al., 2020). However, the CRISPRfinder tool enabled the selection of four candidate sequences, a phenomenon typical of SE genomes (Rossi et al., 2017).

### Comparative genomics

**Figure 2:**
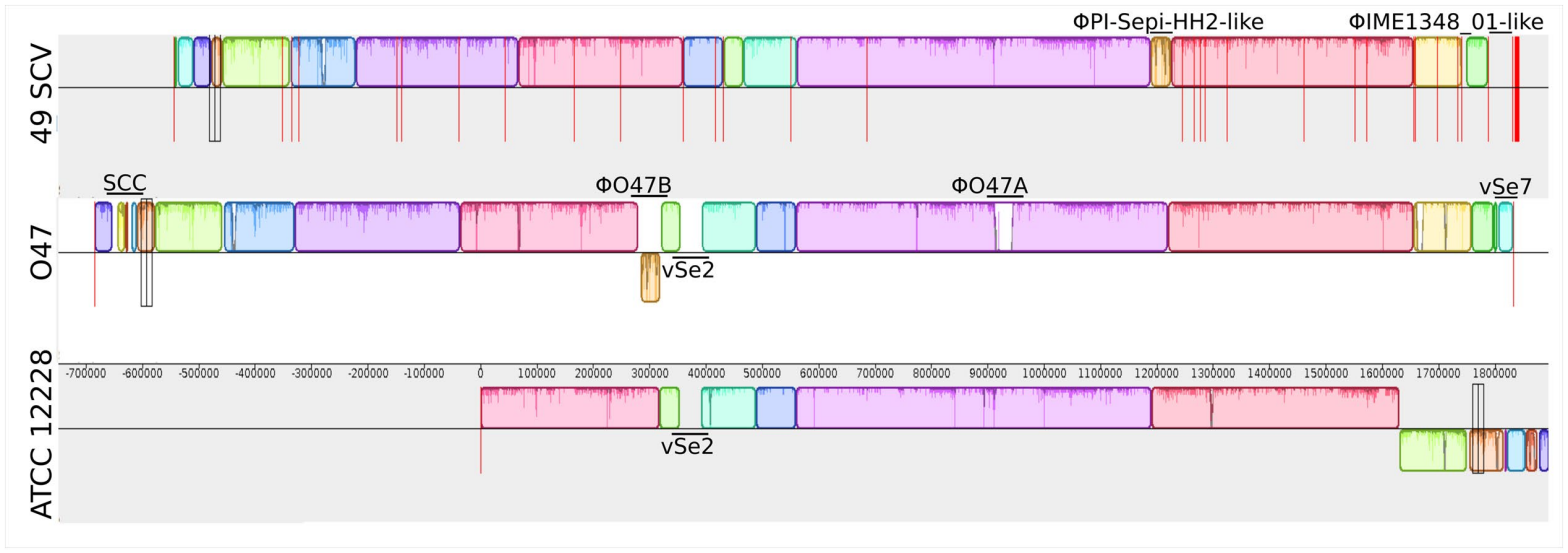
Mauve synteny alignment analysis of 49SCV and the reference O47 and ATCC 12228 genome sequences. Boxes of different colours represent the locally collinear blocks (LCBs). The LCB above the central horizontal line indicates that the sequence direction of the aligned genome is the same as the reference sequence, and the LCB below the reference line indicates that the sequence direction of the aligned genome is opposite to the reference sequence. White areas represent genome-specific sequence elements that may contain genome-specific sequence elements and genomic locations not sufficiently aligned between the selected genomes. Vertical red lines mark the boundaries of the contigs. The Staphylococcal Cassette Chromosome (SCC), genomic islands vSe2 and vSe7, as well as putative prophages (φO47A, φO47B, ΦPI-Sepi-HH2-like, ΦIME1348_01-like) were marked in the individual genomes.

The genomes of 49SCV and the two reference strains O47 and ATCC 12228 were aligned. Visualization of the alignment performed with the Mauve tool is demonstrated in Figure 2. As the 49SCV genome was sequenced in the draft form and available as 39 contigs, it was not possible to draw firm conclusions about the full structure of its genome. However, there were clear blocks of colinearity with the reference genomes, particularly O47, within the contigs. Interestingly, the O47 strain belongs to the same ST (ST2) (Raue et al., 2020) as 49SCV and was originally isolated in an analogous clinical context, that is from a patient with an orthopedic device-associated infection. The most striking differences between the 49SCV and O47 strains included the lack of Staphylococcal Cassette Chromosome (SCC) (see further in the text), the absence of genomic islands such as vSe2 or vSe7, and the lack of a sequence similar to the φO47A prophage in the former strain. It should be noted that a sequence similar to the φO47B was present, but at a different position in the genome and in the opposite orientation (Figure 2).

### Mobile genetic elements and prophages

Using the ISfinder database, we found several sites where mobile elements were present in the genome of 49SCV. It should be noted that the draft genome makes a precise identification of the IS integration sites difficult. This is illustrated by the fact that three contigs in the studied genome [32–34] represent IS sequences (IS1182, IS110, and IS256). In addition, a truncated IS1272 was found in contig 1, between the genes NPW32_RS00680 (encoding a translational GTPase type A) and NPW32_RS00690 (encoding YlaI family protein). In the O47 genome, the IS1272 element was located at the same position. The second site was contig 2, where IS3 was located between the NPW32_RS02965 (*uhpT* gene) and NPW32_RS02975, coding for the SDR family oxidoreductase. The location was analogous to that in O47 (Raue et al., 2020). However, in both cases the transposase component of IS was a non-functional gene (in case of IS1272, it contained a number of nonsense mutations, while in IS3 there was a deletion leading to a frameshift).

Unlike the O47 genome, 49SCV contained a single complex transposon, Tn552 (Rowland and Dyke, 1990). Specifically, the transposon integration site was located within the gene NPW32_RS03445, which encodes a transcriptional regulator of the ArgR family, resulting in its truncation and the appearance of a stop codon after 83 codons. Most notably, the integration resulted in the deletion of a fragment of approximately 12 kbp in size, covering genes FHQ17_RS01225 to FHQ17_RS01275 for the O47 reference. A detailed description of the deleted genes can be found in subsequent sections.

Two prophage sequences were found in the 49SCV genome. One of them was assembled into a separate contig (ΦIME1348_01-like, locus tag: NPW32_RS09955 to NPW32_RS10275, contig 16). The second prophage sequence ΦPI-Sepi-HH2-like was located in contig 1 and was flanked by two genes encoding tRNA (NPW32_RS00090 - tRNA-Asn, NPW32_RS00095 - tRNA-Ser) on one side, and by a truncated gene encoding a plasmid recombination protein (NPW32_RS00320) on the other. Both sequences had a similar size of approximately 38 kbp, allowing them to be classified as *Staphylococcus* class II *Siphoviridae* phages (Deghorain and Van Melderen, 2012). The CheckV tool analysis characterized them as complete, which was consistent with the results described for the phage sequences present in the O47 genome. Blastn search against non-redundant nucleotide database showed that the first prophage sequence (ΦIME1348_01-like) was most similar to bacteriophage IME1348_01 (NC_055036), while the second one (ΦPI-Sepi-HH2-like) was most similar to PI-Sepi-HH2 (MT880871) (Figure 2).

As in other SE genomes, genomic islands were identified in 49SCV. However, only νSeγ, vSe3, and vSe5 islands were present in comparison with the reference genomes. Despite the overall similarity of 49SCV to the O47 genome, vSe2, vSe6, and vSe7 sequences were not identified here and their absence adds to the pool of numerous 49SCV deletions discussed in later sections.

## Streamline of genetic content as a possible strategy associated with the SCV phenotype and its adaptation to chronicity

### 49SCV is devoid of the Staphylococcal Cassette Chromosome (SCC)

**Figure 3:**
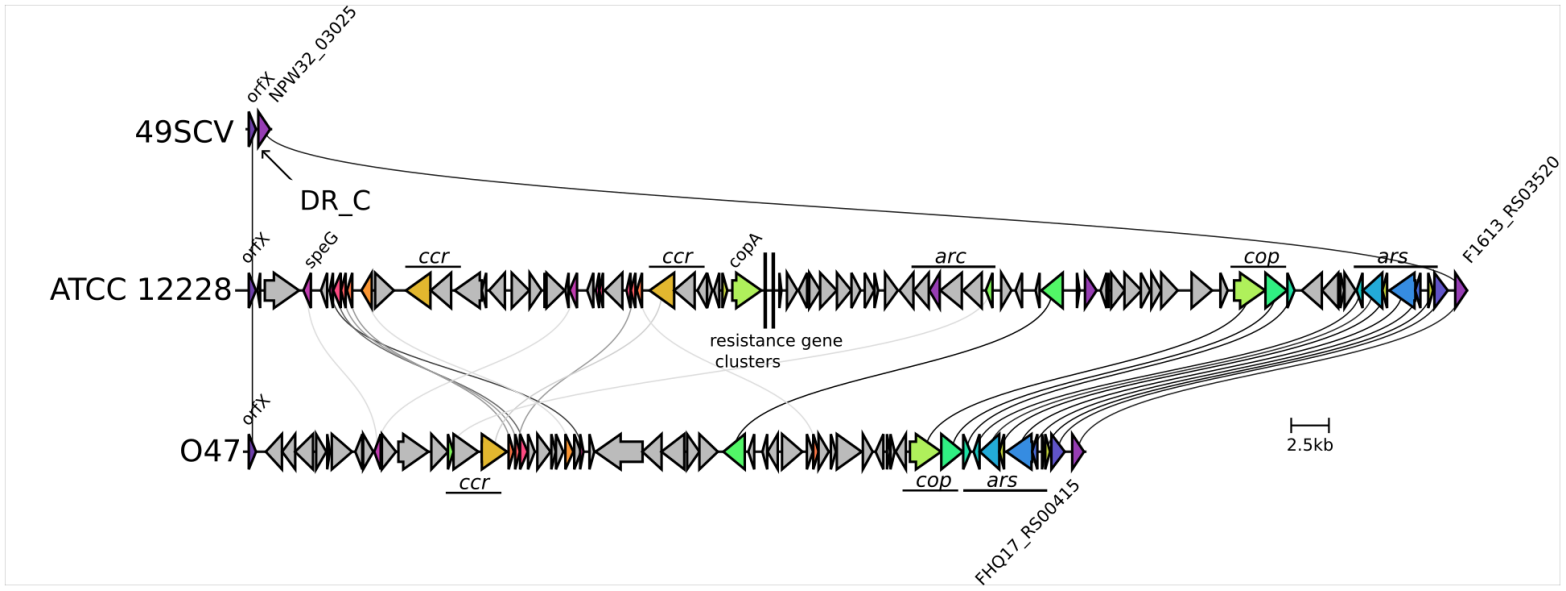
Genetic organisation of the SCC region in 49SCV and in the ATCC 12228 and O47 reference strains. The 49SCV strain is devoid of the SCC element, so only the excision site is marked, flanked on one side by the orfX (with the DR_C sequence marked) and by the NPW32_RS03025 gene on the other side. Sequence similarity of the individual genes greater than 30% was marked with lines connecting the individual CDSs. The same information was expressed by the common colours of the genes in question. In the case of ATCC 12228 and O47 strains significant functional blocks of genes specific to their SCC were also marked. The visualisation was created using the clinker tool.

Comparative genomic analysis revealed the lack of the SCC element in 49SCV which was found in all the analyzed reference genomes (Figure 3). These genomic islands are disseminated among staphylococci and carry antimicrobial resistance and virulence-associated genes (Shore and Coleman, 2013). The most representative SCCs are the SCC*mec* elements harboring the *mecA* or *mecC* genes. These mobile genetic elements constitute a defining feature of methicillin-resistant staphylococci (MRS) which demonstrate a broad-spectrum beta-lactam resistance (Shore and Coleman, 2013; Lakhundi and Zhang, 2018). The SCCs carrying genes other than *mec* have also been identified and reported to harbor diverse genes, including those involved in fusidic acid resistance, capsule synthesis or mercury resistance. The non-*mec* SCCs share mutual characteristics with SCC*mec* by carrying cassette chromosome recombinase (*ccr*) genes in a *ccr* gene complex, integration at a specific integration site sequence (ISS) in the chromosome, and the occurrence of flanking direct repeat containing ISS (Lakhundi and Zhang, 2018). For example, the genome of O47, for which a high level of collinearity with 49SCV was revealed, contains a 54-kb non-*mec* SCC (Raue et al., 2020).

Although the SCC has been considered to play a role in staphylococcal adaptation through the acquisition of additional antimicrobial resistance and virulence genes, its loss can be advantageous, as it may compensate for the fitness cost (Noto et al., 2008). Lin et al. (2016) reported isolation of daptomycin-resistant SA SCVs of the vancomycin-intermediate (VISA) form belonging to the hospital-acquired MRSA lineage during a long-term daptomycin treatment of septic arthritis. In addition to a stepwise development of ultrastructural changes and mutations in SCVs, the loss of SCC*mec* was observed. Conversion to vancomycin resistance has been reported to cause a decrease in the growth rate of MRSA in another study and shown to be partially compensated by the *mecA* deletion. Documented deletions within SCC*mec* during SE infection have also been published (Sendi et al., 2005; Weisser et al., 2010).

### 49SCV demonstrates a ~ 12 kb genomic deletion including the loss of genes involved in the arginine deiminase pathway (ADI)

Genomic comparisons revealed that 49SCV strain was devoid of a ~ 12 kb genomic region present in the analyzed reference strains. The deletion has been substituted with the Tn552 composite transposon. One of the most striking findings highlighting the potential biological significance of this deletion is the consequent loss of genes included in the ADI pathway, which is highly conserved in bacteria and represents a widespread anaerobic route for arginine degradation. The ADI pathway comprises three essential metabolic steps: conversion of L-arginine into L-citrulline and ammonia (1) catalyzed by arginine deiminase (ArcA); transfer of carbamoyl moiety of L-citrulline to phosphate (2) by catabolic ornithine transcarbamylase (ArcB/ArgF) which yields carbamoyl phosphate and L-ornithine; and phosphorylation of ADP (3) by carbamate kinase (ArcC) which occurs with the use of carbamoyl phosphate and results in the production of ATP, carbon dioxide, and ammonia. A key player in the ADI pathway is the L-arginine/L-ornithine antiporter (*arcD* gene) that catalyzes a stoichiometric exchange of one molecule of L-arginine and one molecule of L-ornithine thereby mediating a concomitant uptake of the substrate L-arginine and excretion of the end product L-ornithine. Since metabolic energy is not required for the transport reaction, ATP produced by the ADI pathway can be used for other energy-demanding purposes (Noens and Lolkema, 2017). This metabolic pathway functions as an alternative source of energy for bacterial growth, which is particularly important if neither glucose nor nitrate are available (Makhlin et al., 2007). Moreover, it has been considered a defense mechanism against acidification due to the production of ammonia (Lindgren et al., 2014; Noens and Lolkema, 2017).

Transcription of the *arc* operon is often arginine dependent via the ArgR proteins belonging to the Crp/Fnr-family of transcriptional regulators (Lindgren et al., 2014). Of note, a gene encoding for the ArgR regulator (NPW32_RS03445) has been found truncated in the 49SCV strain and followed by a Tn552-derived sequence (described above). The *argR* homolog was followed by three genes encoding components of the ADI pathway (*arcA*, *argF*, and *arcD*) and a gene encoding for the yet another Crp/Fnr-family transcriptional regulator in all reference SE genomes. Makhlin et al. (2007) demonstrated that transcription of the *arc* operon under anaerobic conditions depends strictly on the functional ArgR (Makhlin et al., 2007). Seggewiss et al. (2006) reported that a hypothetical gene of the Crp/Fnr family (SA2424) being a part of the ADI pathway showed significantly increased transcription in the laboratory-constructed *hemB* mutant of a clinically derived SA SCV strain. Their finding was correlated to up-regulation of the ADI pathway in the mutant strain. The 49SCV apparently lacked the function of both transcriptional regulators of the Crp/Fnr-family.

Genes encoding for enzymes involved in the metabolism of arginine are often duplicated in staphylococci, with a second copy of the *arc* operon contained within the majority of an arginine catabolic mobile element (ACME) allotypes (McManus et al., 2019). ACME is commonly collocated adjacent to SCC*mec* or SCC-associated genes in composite islands. Its carriage has been considered advantageous for bacterial transmission, persistence and survival (McManus et al., 2019). This mobile element was absent in 49SCV which was not surprising due to the lack of the SCC element to which ACME is commonly adjacent. Thus, while one or two genes encoding for the arginine deiminase were identified in the reference strains (core, +/− ACME derived), 49SCV was devoid of any homologous *arcA* genes. Only a residual genetic composition of the ADI pathway was detected in the strain, including the *argF* and *arcC* genes encoding for the ornithine transcarbamylase and carbamate kinase (NPW32_RS02705 and NPW32_RS02710), respectively, and detected elsewhere in the genome.

Our findings indicate the loss of activity of this energy-generating metabolic pathway and stand in contrast to several previous studies. Electron-deficient staphylococcal SCVs (including hemin- and menadione-dependent variants), have been reported to demonstrate an increased expression of the ADI pathway (Kohler et al., 2003, 2008; Seggewiss et al., 2006) as an alternative strategy for ATP production able to compensate for its loss due to defective utilization of a variety of carbon sources, including tricarboxylic cycle intermediates, and electron transport interruption (Kohler et al., 2003, 2008; Kriegeskorte et al., 2014). On the other hand, genes involved in the catabolism of arginine and ornithine were found among the most strongly down-regulated genes in a stable SA SCV strain (exhibiting no classical auxotrophy) isolated from a patient with a PJI relapse (Loss et al., 2019). The 49SCV strain was probably deficient in ATP production not only due to the lack of the functional ADI pathway but also due to a dysfunctional electron transport as it was hemin-dependent. The inhibition of respiratory pathways resulting from mutations in menadione and hemin biosynthesis genes exert physiological effects similar to those experienced in a low-oxygen environment. Since transcriptomic analyses were not performed in our study, we can speculate that other metabolic processes must have provided ATP levels necessary for the strain survival and growth. Accordingly, SCV mutations can be expected to cause upregulation of genes involved in anaerobic metabolism as an adaptive and compensatory response (Cao et al., 2017). Staphylococcal SCVs have been reported to generate ATP mainly from glucose or fructose by substrate phosphorylation which was evidenced by up-regulation of enzymes involved in glycolytic and fermentative pathways (Kohler et al., 2003, 2008; Seggewiss et al., 2006). Additionally, SCVs have been shown to use other optimizing reactions that result in production of ATP including the increased metabolism of glycerol, pyruvate, butanoate/acetoin, and nitrate (Proctor, 2019).

It is tempting to speculate that deletion of specific genetic elements including the SCC cassette and *arc* genes in 49SCV may represent a strategy adopted to streamline the genetic content and improve bacterial ability to infect and survive in the host. According to Both et al. (2021), hyper-variable regions of ACME and SCC*mec* may be a burden in infection and only confer an advantage in some conditions on the human skin as deletions in these regions seemed to occur preferentially in SE strains associated with infection.

## Frameshift-generating events within homopolymeric tracts – phase variation in the background of the SCV phenotype

In order to obtain a complete view of the differences between the 49SCV strain and the reference genomes, especially within the mutated genes, the variant calling against five reference strains using the snippy tool (Seemann, 2023b), which identifies SNPs, MNPs, insertions, deletions and compound mutations was carried out.

**Figure 4:**
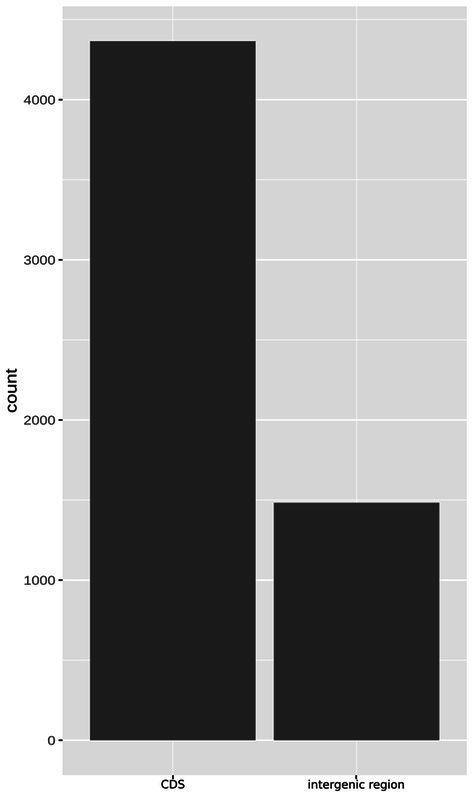
The bar plot demonstrating the abundance of poly(A) and poly(T) homopolymer tracts (HTs), understood as six or more nucleotides of the same type, within gene and intergenic sequences in the genome of 49SCV.

Approximately 200 genes with frameshift mutations were identified in the 49SCV genome. Of note, a large proportion of the frameshift-generating events occurred within specific short sequence repeats (SSRs), namely poly(A) and poly(T) homopolymeric tracts (HTs). The SSRs are highly prone to insertion/deletion (indel) errors due to Slipped-Strand Mispairing (SSM) that occurs during DNA replication or Mismatch Repair (MMR). Variations in the length of SSRs account for one of the three major genetic mechanisms of bacterial phase variation (PV), in addition to the DNA inversion and recombination. The PV, defined as an adaptive mechanism based on the reversible gene expression switch, enables microorganisms to meet the challenge of fluctuating pressures existing in their environment (Gor et al., 2021). Gor et al. found that more than 700 genes in the genome of a highly virulent community-acquired methicillin-resistant SA strain (MW2) contained at least one poly(A) or poly(T) SSR, with a substantial number containing 3–4 SSRs (Gor et al., 2021). In an extensive study of 99 prokaryotic genomes, Orsi et al. (2010) revealed that poly(A) and poly(T) HTs were overrepresented in prokaryotic genes, similarly to our findings. In the analyzed 49SCV genome, 4,365 poly(A) and poly(T) HTs equal to or longer than 6 bp were identified in 1710 coding sequences (CDSs) [2.55 HTs per CD on average], whereas 1,483 of them were localized in intergenic regions (Figure 4).

**Figure 5:**
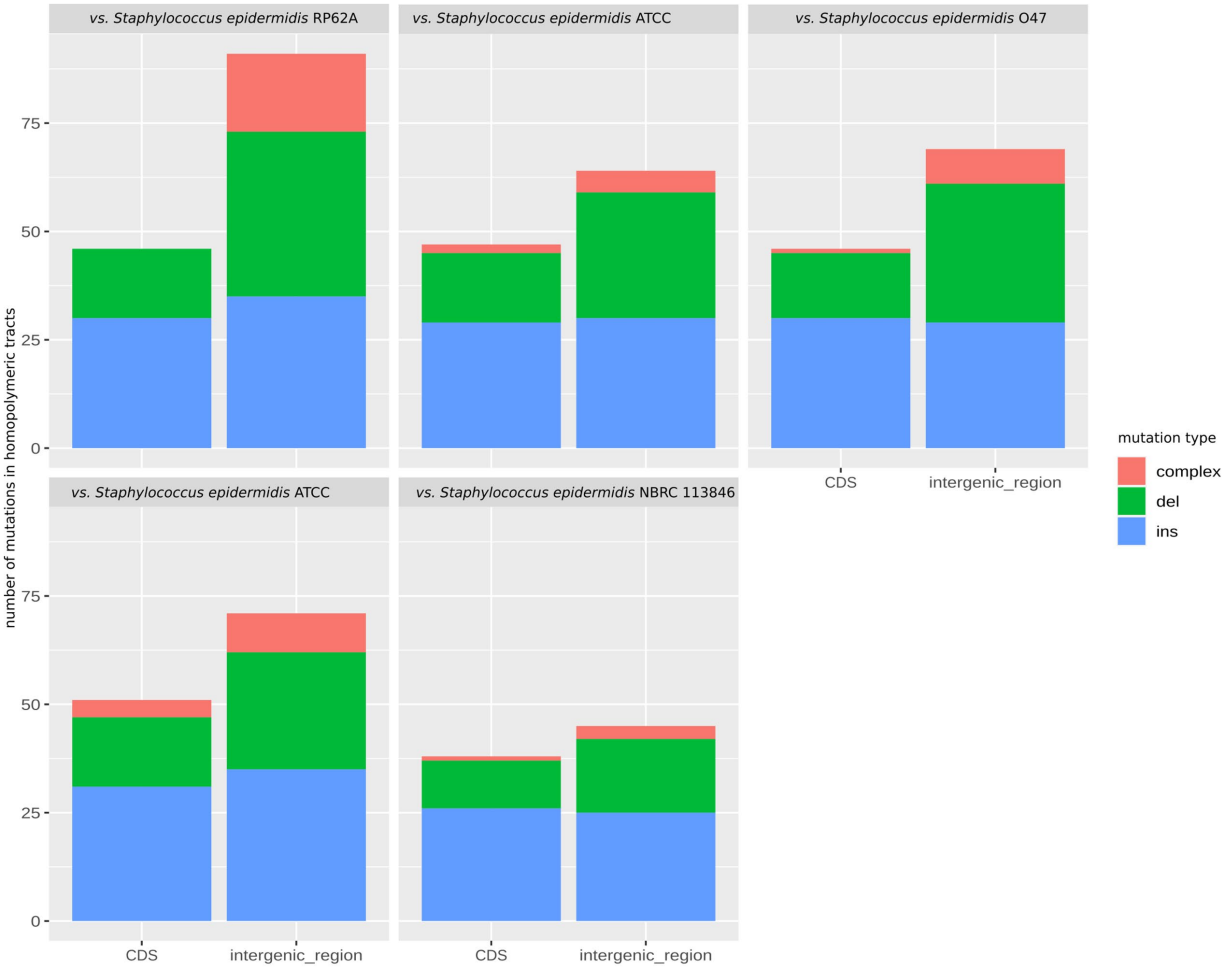
The faceted stacked bar chart showing the number of mutations in the homopolymeric tracts categorized as deletions, insertions, and complex mutations in the genome of 49SCV. The bars represent mutations within coding sequences (CDS) and intergenic regions, while the facets depict mutations observed when comparing with different reference genomes.

While the HTs predominated in the CDSs, indels within the HTs were found predominantly in the intergenic regions in the 49SCV genome, irrespective of the reference genome used for comparison. At the same time, however, 38–51 mutations in CDSs were also identified (Figure 5). Most of these frameshift-generating mutations in the CDSs were insertions, followed by deletions and compound (two different independent changes within one HT) mutations (Figure 5). The predominance of insertions over other types of mutations was less pronounced for intergenic regions (Figure 5). Indels in the SSRs can result in frameshifts that have an ON*↔*OFF effect on gene expression or protein function, depending on the SSR’s location (Gor et al., 2021). If alterations in the length of the SSRs occur in the promoter regions, inhibition of the RNA polymerase binding or a gradation effect on the gene expression can occur (Gor et al., 2021). A shift in the translational reading frame usually results in the synthesis of a truncated and a non-functional protein. In particular, indels at the 5′ end of a coding sequence usually lead to expression of short, non-functional peptides (Orsi et al., 2010). Orsi et al. (2010) revealed the preferential location of HTs at the 5′ ends of genes, indicating selective pressure for the presence of these HTs consistent with their role as regulatory elements. In line with these findings, approximately 70% of indels detected in the poly(A)/poly(T) HTs in 49SCV carried mutations at the 5′-end. Moreover, results of our study revealed that most indels occurred in the poly(A) HTs (Table 1), similarly to the findings of Gor et al. (2021).

**Table 1:** Genes in the 49SCV genome encoding proteins that are truncated due to insertions/deletions within poly(A)/poly(T) tracts and di/tetranucleotide repeats, deletions beyond HTs, internal in-frame deletions or nonsense mutations.

Locus tag 49SCV	Gene name	Truncated/full length* (amino acids)	Homology/function
*-1A in poly(A), frameshift, premature stop codon*			
NPW32_RS09635	*hemA*	406/448	Glutamyl-tRNA reductase/porphyrin-containing compound biosynthetic process
NPW32_RS02415	*ebpS*	279/460 (*additional inframe Ser346_Asp353 deletion)	Elastin-binding protein EbpS/staphylococcal binding to soluble elastin and tropoelastin
NPW32_RS08970	*cls2*	178/494	Phosphatidylserine/phosphatidylglycerophosphate/cardiolipin synthase
NPW32_RS04630	*adhE*	8/869	Bifunctional acetaldehyde-CoA/alcohol dehydrogenase
NPW32_RS08460		152/316	Ornithine cyclodeaminase family protein
*+1A in poly(A), frameshift, premature stop codon*			
NPW32_RS08320	*vraC*	105/120	Hypothetical VraC protein; *vraC* gene is linked to fatty acid biosynthesis genes
NPW32_RS05200	*pxpA*	182/250	5-oxoprolinase subunit PxpA
NPW32_RS03965		96/350	Threonine dehydrogenase-related/zinc-dependent alcohol dehydrogenase family
NPW32_RS09305		81/183	TetR/AcrR family transcriptional regulator (part of the efflux system TetR/EmrA/EmrB)
NPW32_RS09445		39/170	SRPBCC domain-containing protein/activator of Hsp90 ATPase homolog 1-like protein
NPW32_RS05975		424/657	AraC-type regulator; HTH transcriptional regulator
NPW32_RS05815		117/146	MarR family regulator; HTH transcriptional regulator
NPW32_RS08965		190/217	Metal dependent phosphohydrolase with conserved HD motif
NPW32_RS01100	*rsmB*	137/435	16S rRNA methyltransferase RsmB
NPW32_RS00410	*lcpB*	193/412	Anionic cell wall polymer biosynthesis enzyme, LytR-Cps2A-Psr (LCP) family/ ligation of CWGP to PGN
NPW32_RS04140		51/270	MetQ/NlpA family ABC transporter substrate-binding protein
NPW32_RS04645		348/504	ABC transporter permease/substrate-binding protein (fusion)/ transport of one or more from a variety of substrates including glycine betaine and choline
NPW32_RS11435		185/454	PLP-dependent aminotransferase family protein/ DNA-binding transcriptional regulator, MocR family, contains an aminotransferase domain
NPW32_RS09465		174/351	Xaa-Pro aminopeptidase
*-1A in poly(T), frameshift, premature stop codon*			
NPW32_RS03820		40/285	Fructosamine kinase family protein/ deglycation of products of glycation formed from ribose 5-phosphate or erythrose 4-phosphate
*+1A in poly(T), frameshift, premature stop codon*			
NPW32_RS02630		46/413	Major Facilitator Superfamily (MFS) transporter
NPW32_RS06615		24/404	Major Facilitator Superfamily (MFS) transporter
NPW32_RS06735		31/301	Penicillin-binding protein 1B/1F, peptidoglycan transglycosylase/transpeptidase (MrcB)
NPW32_RS00540		75/390	23S rRNA (guanine2069-N7)-methyltransferase RlmK
*Other deletions with frameshift and premature stop*			
NPW32_RS03455	*blaR1*	-1 nt 157/585	Beta-lactam sensor/signal transducer BlaR1
NPW32_RS01930	*phoU1*	-1 nt 7/217	Phosphate signalling complex protein PhoU1
NPW32_RS04840	*efp*	-1 nt 55/185	Translation elongation factor P
NPW32_RS11275	*spxA*	-1(TACG) 118/131	Transcriptional regulator SpxA
NPW32_RS07460		-38 nt 272/305	Phosphatase PAP2 family protein
NPW32_RS05895	*fdhD*	multiple indels and substitutions 6/259	Formate dehydrogenase accessory protein FdhD
*Inframe deletions*			
NPW32_RS03270	*sdrF*	156-aa-deletion in Ser-Asp-rich region at C-terminus 1477/1633	MSCRAMM family adhesin SdrF; Ser-Asp-rich fibrinogen binding protein
*Other insertions with frameshift and premature stop*			
NPW32_RS11105	*gehC*	+1 nt 336/687	YSIRK domain-containing triacylglycerol lipase GehC
NPW32_RS08685	*czrB*	+1(TA) in poli(TA) 72/317	Cation efflux family protein
NPW32_RS05835	*moaA*	2 independent +1A in 2 different poly(A) 111/340333/340	GTP 3′,8-cyclase; conversion of 5’-GTP to 3′,8-cH2GTP
*Nonsense mutations*			
NPW32_RS03860		31/332	Lactate dehydrogenase
NPW32_RS01615	*cls1*	325/490	Phosphatidylserine/phosphatidylglycerophosphate/cardiolipin synthase
NPW32_RS02490		140/252	Short-chain dehydrogenases/reductase (SDR) family
NPW32_RS09180	*rsp*	117/701	AraC family transcriptional regulator Rsp
NPW32_RS09300		214/403	Major Facilitator Superfamily (MFS) transporter
NPW32_RS07065		34/355	Nitric oxide synthase oxygenase (NOS)

The list was prepared using specifically the data from the comparison of the 49SCV genome to the *S. epidermidis* O47 genome. Genes with an annotation ‘hypothetical’ and ‘domain of unknown function (DUF) – containing’, or those identified as pseudogenes in the O47 genome are not included in the table. The complete list of identified mutations can be found in a Supplementary Data File 1. *Length of the homologous protein encoded within O47 genome.

Detection of frameshift-generating mutations within the HTs can suggest an association between PV events and the SCV phenotype. To the best of our knowledge, PV occurring by insertion or deletion of tandem repeats has not been described to occur for staphylococcal SCVs so far. The only investigation linking formation of the SCV phenotype to genetic events reminiscent of the PV phenomena has been published by Kleinert et al. (2017). As evidenced experimentally in their study, transposition and subsequent excision of IS*256* can mediate formation and fast reversion of SA SCV-like phenotypes, respectively. Introduction of IS*256* into a laboratory strain followed by cultivation in the presence of antibiotics resulted in the isolation of SCVs that possessed IS*256* insertions in *guaA* and *hemY* genes and displayed increased resistance to vancomycin and aminoglycosides, respectively.

Genes subject to the PV typically encode cell-surface associated proteins including adhesins and pili but also virulence factors and secreted proteins (Orsi et al., 2010; Gor et al., 2021). It should be noted that indels in the HTs of 49SCV genome occurred in the loci whose biological functions have been reported to be associated with bacterial adherence, metabolism, stress response, virulence, and cell wall synthesis (Table 1). Although we cannot provide the evidence of a direct impact of these genetic events on the expression of proteins, we propose that indels in the HTs within open reading frames (ORFs) can constitute an important background of the SCV phenotype, since a resulting truncation of the corresponding proteins makes their biological dysfunction highly probable.

### Deletion in the poly(A) of the *hemA* gene as a possible trigger factor for phenotype switching

One-nucleotide (1-nt) deletion in the poly(A) HT was detected at the 3′-end of the *hemA* gene (NPW32_RS09635) leading to the generation of a premature stop codon and shortening of the encoded protein by 42 amino acids (Table 1). This gene encodes a glutamyl-tRNA reductase involved in the first step of porphyrin biosynthesis. HemA dimer, which is a probable functional form of the enzyme, forms a complex with glutamate−1-semialdehyde 2,1-aminomutase, the second enzyme in the pathway, suggesting metabolic channeling of a reactive intermediate glutamate-1-semialdehyde. Mutants of *hemA* are dependent on δ-aminolevulinic acid (ALA) for growth and are unable to produce ALA from either glutamate or glutamyl-tRNA (EcoCyc, 2013; Kahl et al., 2016). We hypothesize that truncation of the *hemA* ORF and a consequent synthesis of a dysfunctional HemA protein was a trigger factor for the phenotype switching in 49SCV and a primary reason for the development of hemin auxotrophy in the strain. Functional or genetic defects in the biosynthesis of heme, which is used in the cytochrome biosynthesis, lead to defects in electron transport. As a result, the amount of ATP that is available for the cell wall biosynthesis is drastically reduced. Consequently, electron transport-defective SCVs reveal a lower growth rate and small colonies on agar media (Kahl et al., 2016). Moreover, *hemA* truncation in 49SCV and the subsequent dysfunction of the electron transport system is the most conceivable explanation for a high gentamicin MIC (>256 mg/L) reported previously (Bogut et al., 2014). Uptake of this drug is dependent on the membrane potential, which is significantly reduced when the flow of electrons in the electron transport chain is impaired (Vestergaard et al., 2016). Mutations in the *hemA* gene have been reported in SCVs but only in laboratory-derived mutants of SA. Schaaf et al. characterized genes of the *hem* operon in a stable gentamicin-induced, hemin auxotrophic SA SCV and revealed a deletion in *hemH* and a frameshift at the 3′ end of *hemA*. This frameshift mutation led to truncation of the protein by 54 amino acids (Schaaff et al., 2003). Lannergård et al. (2011) analyzed FusE mutants selected from a drug-susceptible laboratory wild-type SA strain displaying fusidic acid resistance and the SCV phenotype. In addition to mutations in the *rplF* gene (encoding for a ribosomal protein L6), four hemin-auxotrophic strains had missense mutations in *hemA*, *hemB* or *hemH* genes. Truncation of the *hemA* gene in 49SCV is unique due to a deletion in a poly(A) tract. It is worth noting that in comparison to the ortholog from the O47 strain, the *hemA* gene also carried three missense mutations: Ile84Met, Glu89Gln, and Ala357Val (Table 2), which might have further contributed to its dysfunction.

**Table 2:** Genes, belonging to pangenomic gene clusters, for which the predicted amino acid sequence was specific only to the strain 49SCV when compared to the five reference strains.

Locus tag	Gene name	Amino acid change*	Homology/function
NPW32_RS07700		D206V	Multidrug efflux MFS transporter
NPW32_RS02785		F29L	MFS transporter
NPW32_RS05430	*sdrM*	A312G	Multidrug efflux MFS transporter SdrM
NPW32_RS10830	*yjbH*	G93S	Protease adaptor protein YjbH
NPW32_RS07465	*menH*	R70K, E252K	2-succinyl-6-hydroxy-2,4-cyclohexadiene-1-carboxylate synthase MenH/menaquinone biosynthesis
NPW32_RS00355	*menA*	A108V	1,4-dihydroxy-2-naphthoate polyprenyltransferase MenA/menaquinone biosynthesis
NPW32_RS05115	*hemN*(*hemW*)	P205L	Oxygen-independent coproporphyrinogen III oxidase/heme biosynthesis
NPW32_RS07655		A155T	Sensor histidine kinase LytS/YehU family
NPW32_RS05480		L59P, A81V	Alanine/ornithine racemase family PLP-dependent enzyme
NPW32_RS03325	*secA2*	T108A	Accessory Sec system translocase SecA2
NPW32_RS07960	*graX*/*apsX*	A30T	Nucleoside-diphosphate-sugar epimerase (WcaG)/auxiliary protein GraX/ApsX/cell wall/membrane/envelope biogenesis
NPW32_RS09060	*sigB*	M65I	RNA polymerase sigma factor SigB
NPW32_RS01410		L62F	Predicted Zn-dependent peptidase, M16 family (PqqL)
NPW32_RS07210	*vraR*	A202V (8th aa from the C-terminus)	Three-component system response regulator VraR
NPW32_RS04480	*secA*	P656T	Preprotein translocase subunit SecA
NPW32_RS06635		V283M	Flavoprotein YhiN/ NAD(P)/FAD-dependent oxidoreductase
NPW32_RS00235**	*clpP*	V24I, K52E, S138A, F175V, N218T, N227S, N228D	Clp protease ClpP
NPW32_RS11240		G430V	Predicted extracellular nuclease/GldG family
NPW32_RS03030	*rlmH*	H158R (2nd aa from the C-terminus)	23S rRNA pseudouridine(1915)-N(3))-methyltransferase RlmH
NPW32_RS10795		K76E, P125S, L152F	Predicted N-acetyltransferase YhbS
NPW32_RS06905***		T64I, A109V, S117F, L149F, L184M, S359A, N370D	Sensor histidine kinase YesM/two-component system, LytTR family, sensor histidine kinase AgrC /GHKL domain-containing protein
NPW32_RS09140	*nreC*	V118A	Nitrate respiration regulation response regulator NreC

*Missense substitutions were indicated if in the compared group only 49SCV was different then reference strains. **Only 49SCV and O47 in a cluster. ***Cluster lacking O47 sequence.

### Other mutations within the HTs and beyond them – their possible association with the SCV phenotype

#### Genes associated with adhesion and growth rate

Staphylococci possess a wide range of structurally-related surface proteins with adhesive properties and many of them belong to a structurally related family of microbial surface components recognizing adhesive matrix molecules (MSCRAMMs), that facilitate colonization step in the pathogenesis of prosthetic device infections (Arrecubieta et al., 2009). Two genes encoding for the MSCRAMMs (*ebpS, sdrF*) have been found truncated in 49SCV which can suggest an association with the strain phenotype.

A conservative inframe deletion of an internal gene fragment encoding 156 alternating amino acid residues in a specific, C-terminal Ser-Asp-rich domain was identified in the *sdrF* gene (NPW32_RS03270). SdrF produced by SE binds collagen and has been reported to play a significant role in the initiation of ventricular assist device driveline infections (Arrecubieta et al., 2009). Another work reported the involvement of SdrF in the adherence to human keratin and suggested its role in facilitating skin colonization by SE (Trivedi et al., 2017). Hence, its role in SE pathogenesis remains to be elucidated.

Genetic changes in the *ebpS* gene (NPW32_RS02415) encoding an elastin-binding protein (EbpS) included a conservative inframe deletion of several codons and one-nucleotide deletion in the poly(A) resulting in shortening of the protein by 181 amino acids (Table 1). We assume that accumulation of mutations in this gene might have had a potential biological significance. The EbpS protein has been reported to mediate staphylococcal binding to soluble elastin and tropoelastin (Campoccia et al., 2009) but one of the previous studies demonstrated that the adherence to immobilized elastin in SA is rather mediated by fibronectin-binding proteins, and inactivation of the *ebpS* has a minimal effect on bacterial binding to elastin peptides (Roche et al., 2004). Interestingly, Campoccia et al. (2009) identified a group of new variant forms of the *ebpS* gene in SA clinical strains isolated from implant-related orthopedic infections which were shortened for 180 bp. The corresponding protein amino acid sequence lacked an entire peptide segment of 60 amino acids implicating the disappearance of its entire hydrophobic domain (Campoccia et al., 2009). Nakakido et al. (2014) suggested that despite its relatively weak contribution to adhesion, EbpS may play other important roles including the regulation of the biofilm formation that are zinc concentration dependent. Importantly, the authors observed a considerably decreased growth rate of the EbpS-deficient mutant strain. Similarly, the EbpS expression was correlated with the ability of cells to grow to a higher density in liquid culture in another study, implying its role in regulating cell growth (Downer et al., 2002). It can be hypothesized that truncation of the EbpS protein might have an association with a slowly growing phenotype of 49SCV. One of the most recent works suggested that downregulation of genes encoding surface proteins can be beneficial for immune evasion and survival of SCVs due to reduced adhesion to phagocytic cells (Liu et al., 2023). It can support our assumptions regarding the possible involvement of the *ebpS* and *sdrF* mutations in the SCV phenotype.

The elongation factor P (EF-P) is a universally conserved translation factor that alleviates ribosome pausing at polyproline motifs by facilitating peptide bond formation. In the absence of EF-P, a polyproline peptide bond formation can limit translation rate, leading to pleiotropic phenotypes including slowed growth, increased antibiotic sensitivity, and the loss of virulence (Balibar et al., 2013; Tollerson et al., 2018). The *efp* gene has been classified among genes important for survival and growth of SA (Chaudhuri et al., 2009). The *efp* ortholog in 49SCV (NPW32_RS04840) was found to carry 1-nt deletion resulting in a frameshift and protein shortening by 130 aa which can suggest a loss-of-function effect. Due to the critical role of EF-P in cell growth and virulence its truncation could have been involved in the 49SCV phenotype.

The *pxpA* homolog in NPW32_RS05200 locus encoded a protein truncated by 68aa when compared to its homolog in the genome of the O47 strain. The *pxpA*-encoded 5-oxoprolinase catalyzes the cleavage of 5-Oxo-L-proline (OP) to form L-glutamate which is coupled to the hydrolysis of ATP to ADP and inorganic phosphate (UniProtKB|UniProt, n.d.). The OP is considered one of the major metabolite damage products. Its accumulation is deleterious and it can lead to a bacterial growth inhibition (Park et al., 2001). In the study of Niehaus et al. inactivation of *pxpA*, *pxpB*, or *pxpC* genes in *Bacillus subtilis* slowed growth, caused OP accumulation in cells and medium, and prevented its use as a nitrogen source (Niehaus et al., 2017). These results are suggestive of a possible contribution of the *pxpA* gene truncation to the slowed growth of 49SCV.

#### Genes associated with the cell membrane and cell wall composition

Genetic events that might have led to dysfunction of the corresponding proteins were detected in two cardiolipin (CL) synthase genes, namely *cls1* and *cls2*. The NPW32_RS08970 locus was found to carry a *cls2* homolog encoding a truncated protein due to a deletion in the poly(A) HT which generated a frameshift and a premature stop codon (178 aa/494 aa). The other gene (NPW32_RS01615) – *cls1* homolog - encoded a truncated protein variant due to a nonsense mutation (325aa/490aa) (Table 1). The CL synthase mediates condensation of two phosphatidylglycerol (PG) molecules to yield cardiolipin and glycerol. The phospholipid composition of bacterial membranes undergoes changes triggered by the growth phase or environmental stressors. In actively growing SA, the predominant phospholipid is PG, whereas CL becomes predominant in a stationary growth phase, with a corresponding decline in the PG content. Similar changes have been observed under conditions of osmotic stress, energy deprivation, or after phagocytosis by polymorphonuclear leukocytes (PMN) (Koprivnjak et al., 2011; Ohniwa et al., 2013). Both *cls1* and *cls2* participate in the CL accumulation in SA, where the *cls2* gene serves a housekeeping function and the *cls1* is active under stress conditions including high salinity or low pH (Tsai et al., 2011; Ohniwa et al., 2013). Deletion of both *cls* genes was found to abolish the CL synthesis (Koprivnjak et al., 2011; Tsai et al., 2011). Membrane composition can be modulated and other components such as the PG can compensate for the stalled function of the *cls* genes, which might have been the case for the 49SCV strain. The PG level was observed to be increased in mutants that did not accumulate CL (Tsai et al., 2011).

The *vraC* gene homolog in NPW32_RS08320 locus encodes a protein truncated by 15C-terminal amino acid residues (Table 1). The gene is a part of the cluster of five genes transcribed in the same direction: the gene encoding a HAD family hydrolase (NPW32_RS08335), *vraA* encoding a long chain fatty acid CoA ligase or acyl-CoA synthetase (EC 6.2.1.3; NPW32_RS08330), *vraB* encoding an acetyl-CoA C-acetyltransferase (EC 2.3.1.9; NPW32_RS08325), *vraC* (with no particular annotation), and a gene encoding a hypothetical protein (NPW32_RS08315). This gene cluster is highly syntenic in staphylococci, and proteins encoded in NPW32_RS08320 - NPW32_RS08315 loci are reminiscent of the recently described VraC and VraP proteins (Wang and Sun, 2021). One study showed that *vraC* was co-transcribed with *vraP* located downstream of *vraC* and that encoded proteins may form a stable complex to play a key role in the cell wall metabolism. VraCP promoted the expression of the cell wall synthesis (*glyS, sgtB, ddl* and *alr2*) and hydrolysis (*sceD*, *lytM* and *isaA*) genes which influenced the cell wall thickness, antibiotic resistance and autolysis rate. Deletion of *vraC, vraP* and *vraCP* led to phenotypic alterations, including increased susceptibility to the cell wall-associated antibiotics, reduced cell wall thickness and decreased autolysis (Wang and Sun, 2021). It can, therefore, be assumed that VraC truncation in 49SCV could have played a role in the cell wall remodeling reported previously for SCVs (Onyango et al., 2013).

A hypothetical threonine dehydrogenase/zinc-dependent alcohol dehydrogenase family encoding gene in the NPW32_RS03965 locus was found disrupted by insertion in the poly(A) leading to truncation of a resultant protein (96aa/350aa) (Table 1). The enzyme L-threonine dehydrogenase was shown to be the first enzyme of the pathway converting threonine to glycine (Newman et al., 1976). The dehydrogenase pathway degrades threonine in two steps to acetyl-CoA and glycine. Acetyl-CoA is required for carbon source utilization, whereas glycine and its subsequent degradation are required for nitrogen source utilization (Reitzer, 2014). Glycine also represents the principal ingredient of bridges linking peptidoglycan units in staphylococci (Liu et al., 2021). Liu et al. (2021) reported that genetic mutations in a stable catheter-derived SE SCV were detected in genes involved in an array of cellular metabolic processes including glycine and threonine metabolism. While contents of most detected amino acids increased significantly in the SCV strain, glycine demonstrated a significantly lower level. The reduced glycine concentration was interlinked with the frameshift in *SERP1287* gene, whose product is the alanine-glyoxylate aminotransferase (AGXT1) that catalyzes synthesis of glycine from alanine or glyoxylate (Liu et al., 2021). Their results make a potential association between the frameshift-generated truncation of the threonine dehydrogenase and dysfunction of one of the pathways of glycin biosynthesis in 49SCV conceivable. The resultant shortage in glycine might, in turn, have a negative influence on the growth of the strain by decreasing the peptidoglycan-linking rate.

It was also revealed that 49SCV harbored three *lcp* genes (NPW32_RS01825, NPW32_RS06035 and NPW32_RS00410 loci encoding for LcpA, LcpC, and LcpB proteins, respectively) whose products are essential for the optimal cell separation. The *lcp*-encoded LytR-CpsA-Psr (LCP) transferases are involved in the final ligation step of the cell wall glycopolymers to the peptidoglycan backbone (Stefanović et al., 2021). The *lcpB* gene had an insertion within the poly(A) resulting in frameshifting and the protein truncation (193aa/412aa). Dysfunctional LcpB could have been linked to the SCV phenotype and impaired growth rate. Following characterization of single-, double- and triple-deletion *lcp* mutants in SA, distinct phenotypes for each of the three proteins were suggested, including LcpA as the one involved in cell separation and septum formation, LcpB protecting cells from autolysis, and LcpC enhancing the properties of *lcpA* and *lcpB* mutants when deleted (Stefanović et al., 2021). Deletion of all three LCP genes resulted in complete teichoic acid loss in staphylococcal cell walls whereas deletion of any of individual LCP genes disturbed their attachment (Chan et al., 2013). Previous studies showed that the loss of one or more of the *lcp* genes can be interlinked with an aberrant septum and biofilm formation, increased susceptibility to β-lactam antibiotics, autolysis, induction of the cell wall stress responses, and reduced levels of envelope phosphate (Hübscher et al., 2009; Over et al., 2011; Dengler et al., 2012; Chan et al., 2013). A severely defective growth phenotype was observed in the triple *lcp* mutant. On the other hand, bacterial growth could be rescued to varying degrees by any one of the three proteins, suggesting some functional redundancy and flexibility in maintaining cell division (Over et al., 2011).

Undecaprenyl phosphate (UP) is an essential compound in the biosynthesis of bacterial extracellular polysaccharides including peptidoglycan and teichoic acids. It is produced by the dephosphorylation of undecaprenyl diphosphate (UPP) via *de novo* synthetic and recycling pathways. Gram-positive bacteria contain remarkable amounts of undecaprenol, which is phosphorylated to UP. Dephosphorylation of UPP is catalyzed by the BacA homolog and the type-2 phosphatidic acid phosphatase (PAP2) homolog. The presence of one of these UPP phosphatases is essential for bacterial growth (Kawakami and Fujisaki, 2018). The 49SCV strain had three genes annotated as the *pap2* homologs (NPW32_RS02135, NPW32_RS07460, NPW32_RS10675) among which NPW32_RS07460 showed a deletion resulting in protein truncation by 23 amino acids. Dysfunctional protein might have led to impairment of the cell wall synthesis in the strain.

Spherical cocci synthesize peptidoglycan at the septum only. This process involves two essential penicillin-binding proteins (PBPs), namely PBP1 (transpeptidase) and a bifunctional PBP2 (transpeptidase and transglycosylase) (Pinho et al., 2013). The NPW32_RS06735 locus in 49SCV encoded a protein with a similarity to penicillin-binding proteins 1B/1F and peptidoglycan transglycosylase/transpeptidase family (MrcB). It can be speculated that a substantial shortening (31 aa/301 aa) of this protein due to a 1 nt insertion in the poly(T) and a premature stop codon might have had an effect on the peptidoglycan synthesis and cell division in this strain. One of the previous studies showed that the loss of PBP1 or its C-terminal domains, which can bind peptidoglycan and potentially coordinate the cell division process, result in cessation of division at the point of septal plate formation. The loss of PBP1 transpeptidase activity was linked to a phenotype of thickened and aberrant septa (Wacnik et al., 2022).

#### Genes associated with virulence

A 1-nt insertion resulting in the frameshift in the *gehC* gene (NPW32_RS11105) was detected in 49SCV. The gene encodes the YSIRK domain-containing triacylglycerol lipase. The insertion led to a premature codon and the protein truncation (336 aa/687 aa). It was previously reported (Longshaw et al., 2000) that, in addition to GehC, SE can produce a second lipase, designated GehD. Both genes were found present in isolates from both clinical and non-clinical backgrounds. Moreover, GehD could partially compensate for the GehC enzyme in a laboratory-derived mutant. The GehC shortening resulting in a loss of lipase activity in the O47 strain was reported in the study of Raue et al. (2020). Since 49SCV encoded a truncated, and most probably dysfunctional GehC protein, it is conceivable that its virulence related to the lipase activity could have been reduced, even if partially present due to an intact *gehD* gene (NPW32_RS02885).

Xaa-Pro aminopeptidases, enzymes widely distributed in bacteria, catalyze the release of dipeptides from the amino termini of polypeptides containing a proline or an alanine at the penultimate position (Ge et al., 2009). A presumed Xaa-Pro aminopeptidase has been recently reported as a novel virulence determinant in *Campylobacter jejuni*. Its deficiency in the mutant strain corresponded to a significantly lower virulence in a murine model of campylobacteriosis (Heimesaat et al., 2020). Xaa-Pro aminopeptidases have also been reported in *E. coli* (Zheng et al., 2005), streptococci (Goldstein et al., 2001; De et al., 2016) or *Porphyromonas gingivalis* (Yagishita et al., 2001) and linked to their virulence. The NPW32_RS09465 gene in 49SCV was annotated as the Xaa-Pro aminopeptidase and appeared to be disturbed by insertion in the poly(A) HT leading to the protein truncation (174 aa/ 351 aa) and dysfunction. No other gene encoding a homologous protein was found in the 49SCV genome, suggesting that dysfunction of the NPW32_RS09465 may be linked with a decreased virulence.

#### Genes associated with metabolism

Deletion of adenine in the poly(A) HT leading to a premature stop codon (8aa/869aa) was identified in the *adhE* gene (synonymous gene name *adhC*; NPW32_RS04630) encoding a hypothetical bifunctional acetaldehyde-CoA dehydrogenase and an iron-dependent alcohol dehydrogenase. The AdhE catalyzes the sequential reduction of acetyl-CoA to acetaldehyde and then to ethanol under fermentative conditions (EcoCyc, 2022). Additionally, a nonsense mutation detected in the NPW32_RS03860 locus, leading to truncation (31aa/332aa) of the corresponding D-lactate dehydrogenase enzyme, allowed us to hypothesize that a possible dysfunction of the two enzymes could have been related to redirection of the 49SCV metabolic activity.

Under growth-limiting conditions that exist during an infectious process, coupled with an inability of electron transport-deficient SCVs to produce energy via oxidative phosphorylation, ATP production can be sustained by the substrate level phosphorylation. It was previously reported in the (Kohler et al., 2003) that proteins involved in the glycolytic and fermentation pathways were increased in all phases of growth of the *hemB* mutant SCV of SA. Alcohol dehydrogenase (Adh) and L-lactate dehydrogenase (L-Ldh) were reported among proteins induced by the *hemB* mutation with lactate being the main fermentation product. Since mutations were detected in the aforementioned loci encoding for the Adh and Ldh enzymes in the genome of 49SCV, it can be assumed that either their possible dysfunction could have been compensated by the corresponding enzymes encoded by alternative genes or that metabolic pathways other than those based on the ethanol or lactate production could have been implicated in the metabolism of the strain. Interestingly, Kriegeskorte et al. (2014) reported that, in contrast to the *hemB* mutant and gentamicin-induced SCVs of SA, clinically derived SCVs showed no prominent up-regulation of glycolytic proteins.

Another deletion was detected in the poly(A) HT of the NPW32_RS08460 locus encoding for a hypothetical ornithine cyclodeaminase/μ-crystallin (OCD/CRYM) family protein. The OCD/CRYM superfamily consists of enzymes involved in the metabolism of amino acids (Uma Mahesh et al., 2022). Since the enzymes are functionally diverse and the gene annotation in 49SCV was not sufficient to identify the exact enzyme and its biological function, it is impossible to predict the possible effect of this hypothetical protein truncation (152aa/316aa) on the metabolism of the strain.

Similarly, a nonsense mutation detected in the NPW32_RS02490 locus of 49SCV leading to truncation (140aa/252aa) of the corresponding protein makes speculations about its probable dysfunction conceivable but impossible to link to any specific metabolic process. The protein is a member of a large NAD(P)(H)-dependent short-chain dehydrogenases/reductase (SDR) family. The family includes enzymes that catalyze reactions on a wide range of substrates and play critical roles in lipid, amino acid, carbohydrate, cofactor, and xenobiotic metabolism as well as in redox sensor mechanisms (Lord et al., 2014).

#### Genes encoding transcriptional regulators

Rapid, adaptive responses triggered by regulatory proteins are crucial for bacterial survival under challenging conditions existing in the host. They respond to specific environmental and cellular signals leading to modulation of transcription, translation, or some other events in gene expression. As a result, physiological responses are modified appropriately (Ramos et al., 2005). The most abundant groups of transcriptional regulators encoded in bacterial genomes include the LysR, TetR/AcrR, AraC/XylS, and Lrp families (Kotecka et al., 2021).

The NPW32_RS09305 locus of 49SCV harbored a gene encoding a hypothetical protein with TetR/AcrR homology. An insertion detected in the poly(A) of this gene was associated with a substantial truncation of the ORF. Interestingly, the gene in the adjacent locus NPW32_RS09300, encoding a hypothetical MFS (Major Facilitator Superfamily) transporter, was also disrupted due to a nonsense mutation leading to the protein truncation (Table 1). The TetR/AcrR family proteins generally function as transcriptional repressors and regulate a wide range of cellular activities, including osmotic stress, homeostasis, biosynthesis of antibiotics, multidrug resistance, efflux pumps, enzymes implicated in different catabolic pathways, virulence and pathogenicity of bacteria (Ramos et al., 2005; Deng et al., 2013). Prediction on the role of TetR/AcrR in 49SCV will not be possible without further genetic experiments and metabolic modelling.

Insertion in the poly(A) leading to truncation of the encoded protein was also detected in the locus NPW32_RS05975 annotated as the AraC-type transcriptional regulator. The AraC family regulators mostly act as activators of gene expression in bacteria. They control diverse cellular functions including carbon metabolism, type III secretion systems, stress response, quorum sensing and virulence (Kotecka et al., 2021). For example, AraC-type transcriptional regulator Rbf was found to promote the *icaADBC* operon expression by a negative regulation of expression of *sarR* encoding for the *ica* operon repressor. The Rbf regulator was also able to indirectly repress the *ica* operon activator, SarX. A complicated interplay between Rbf and the two Sar family proteins in the regulation of the biofilm phenotype has been suggested. However, in the hierarchy of biofilm regulators, IcaR was found dominant over the Rbf-SarR-SarX axis (Rowe et al., 2016). The NPW32_RS05975 locus in 49SCV was found adjacent to genes encoding a hypothetical transcriptional regulator (NPW32_RS05970), a hypothetical PH domain-containing protein (NPW32_RS05965), and SarR transcriptional regulator (subfamily within SarA-family; NPW32_RS05960). The gene encoding a global transcriptional regulator SarA itself was located elsewhere in the genome (NPW32_RS07800). The NPW32_RS05975 encoded protein was only 22%/44% identical/similar to Rbf encoded by SA0622 in *Staphylococcus aureus* subsp. *aureus* N315. However, there was another gene encoding an AraC-type transcriptional regulator in 49SCV. Similarity/identity of NPW32_RS08005-encoded protein to Rbf was 49%/68%, suggesting its possible role as a functional homolog. Altogether, the Rbf regulatory function did not seem to be affected in 49SCV.

Li et al. (2015) reported that the AraC-type transcriptional regulator, Rsp, promotes the production of key toxins while repressing major biofilm-associated genes and biofilm formation in SA. Their study indicated that upregulation of the accessory gene regulator (Agr) and downregulation of the *ica* operon were central to the regulatory impact of Rsp on virulence. The Rsp protein was found to directly bind to the *agrP2* and *icaADBC* promoters, resulting in increased levels of the Agr-controlled toxins, phenol-soluble modulins (PSMs) and alpha-toxin, as well as a reduced production of PIA. The Rsp can, therefore, be regarded as an essential regulator for the development of acute SA infections. Its role in the regulation of gene expression in SE remains unsolved but a truncation of the protein encoded by the *rsp* ortholog in 49SCV (NPW32_RS09180) due to a nonsense mutation was suggestive of the Rsp protein dysfunction, which might have been correlated to the PIA-mediated proficient biofilm production.

Additionally, a deletion of one out of the two tetranucleotides TACG in the *spxA* gene (NPW32_RS11275) was observed in 49SCV. The encoded protein has been annotated as a negative transcriptional regulator of the biofilm formation in SE. It affects the PIA production by regulating transcription of the *icaADBC* in an *icaR*-independent manner. ClpP protease has also been involved in this process as evidenced by its ability to degrade Spx and enhance biofilm formation (Wang et al., 2010). Since the *ica*-positive 49SCV strain was reported as a proficient biofilm producer under *in vitro* conditions (Bogut et al., 2014), inactivity of this regulatory pathway is conceivable, taking into account genetic changes detected both in the *spxA* (truncation of the protein by 13 amino acids) and *clpP* (missense mutations) genes (Table 1).

The 1-nt insertion was also detected in the poly(A) HT in the NPW32_RS11435 locus, generating frameshift and a premature stop codon (185aa/454aa) in the gene encoding the PLP (pyridoxal 5′-phosphate - a biologically active form of vitamin B6) - dependent aminotransferase family protein/ DNA-binding transcriptional regulator belonging to the MocR family. The MocR-like transcription factors (MocR-TFs) represent a group of understudied chimeric proteins formed by the fusion between DNA-binding proteins and PLP-dependent enzymes (Tramonti et al., 2018). The MocR-TFs subfamily was predicted to control transcription of bacterial genes involved in diverse processes including vitamin B6, gamma aminobutyric acid (GABA), taurine, and ectoine metabolism. MocR-TF regulators were also suggested to control genes encoding enzymes involved in reduction/oxidation processes, various transporters and PLP-dependent enzymes (Suvorova and Rodionov, 2016). Genomic analyses demonstrated that MocR-TFs are widespread among eubacteria, implying their essential role in the metabolism but knowledge on these regulators is still scarce as only few of them have been experimentally characterized (Tramonti et al., 2018). Prediction on the role of MocR-TF in 49SCV will not be possible without further genetic experiments and metabolic modelling.

#### Genes encoding for transport system proteins

The disrupted [1 nt insertion in the poly(A)] gene encoding a truncated (51 aa/270 aa) MetQ/NlpA family ABC transporter substrate-binding protein (NPW32_RS04140) was located downstream of the NPW32_RS04145 (encoding an ABC transporter permease), and NPW32_RS04150 (encoding a methionine ABC transporter ATP-binding protein) loci. All three genes are transcribed in the same direction and most probably make up an operon. Interestingly, a regulatory S-adenosylmethionine-binding riboswitch class I sequence was predicted upstream of the first NPW32_RS4150 gene. The hypothetical protein encoded by the disrupted NPW32_RS04140 gene demonstrates characteristics of lipoproteins. *In silico* analyses with tools devoted to lipoprotein prediction (LipoP for Gram-negative and PRED-LIPO for Gram-positive bacteria) revealed the presence of a lipoprotein signal sequence with a cleavage site LAA(19)↓C(20)G. Methionine plays a key role in the protein biosynthesis initiation and many cellular processes with a stabilizing role in protein structure (Beavers et al., 2021). At least two transport systems can serve for the entry of methionine into *E. coli* (Kadner and Watson, 1974), and there was another possible methionine transport system in 49SCV encoded by the genes: NPW32_RS10720 (*gmpC*) annotated as a dipeptide ABC transporter glycylmethionine-binding lipoprotein, NPW32_RS10715, encoding an ABC transporter permease, and NPW32_RS10710 annotated as a methionine ABC transporter ATP-binding protein. Given that many bacteria including staphylococci have evolved mechanisms to synthesize methionine *de novo* due to its importance for viability (Schoenfelder et al., 2013) and that the second methionine transport system that can compensate for dysfunction of the other was identified, the negative impact of the mutation in NPW32_RS04140 in 49SCV is questionable.

Another transport system whose element was found truncated in 49SCV was the ABC transporter permease/substrate-binding protein (fused permease and substrate binding protein) involved in the transport of one or more from a variety of substrates including glycine betaine and choline (NPW32_RS04645). The ABC transporters are multisubunit complexes composed of integral membrane proteins that function as a permease, peripheral membrane ATP binding proteins able to hydrolyze ATP, and extracellular substrate binding proteins (SBPs) acting as receptors for the substrate to be transported. Although structurally conserved, these transporters play a role in the uptake of a diverse range of molecules (Williams et al., 2004). Choline and glycine betaine were reported to act as osmoprotectants enhancing staphylococcal growth at high osmolarity (Graham and Wilkinson, 1992). Stimeling et al. (1994) provided evidence for two glycine betaine transport systems in SA which cannot exclude their mutually compensatory roles. Similarly, another gene with annotation as encoding a betaine/proline/choline family ABC transporter ATP-binding protein (NPW32_RS02740; contig 2) was identified in the 49SCV genome. Hence, effective osmoprotection occuring in SCVs including 49SCV in spite of a truncated osmoprotectant transport system cannot be ruled out.

We also identified three genes encoding for hypothetical Major Facilitator Superfamily (MFS) proteins to be disrupted either by insertion in poly(T) (NPW32_RS02630, NPW32_RS06615) or through a nonsense mutation (NPW32_RS09300). However, lack of specific annotation or similarity unables to comment on the possible contribution of these mutations to 49SCV phenotype.

#### Genes associated with stress response

Fructosamine kinase family (FN3K) comprises proteins involved in the removal of fructosamines produced during a spontaneous reaction of glucose with amines. As a result, FN3K protects proteins against damage caused by high glucose concentrations. Its translation in SA was reported to remain under control of RsaI - a small non-coding RNA. Following glucose consumption, translation of permease involved in the uptake of glucose and the FN3K enzyme is repressed by RsaI (Bronesky et al., 2019). A possible dysfunction of FN3K enzyme in 49SCV (NPW32_RS03820) caused by a one-nucleotide deletion within the gene poly(T) HT and truncation of the resultant protein may suggest the existence of genetically based metabolic disturbances in terms of glucose processing that might have had influence on its slow growth. On the other hand, it may seem conceivable that truncation of the protein that plays its detoxification role at high glucose concentrations may represent one of the possible ways to streamline genetic content to adapt the microorganism to nutrient deficiency in the depths of the implant-associated biofilm structure.

The 49SCV genome also harbored a 2 nt insertion in the poli(TA) of the *czrB* (NPW32_RS08685) gene leading to truncation of the corresponding protein (72 aa/317 aa). The *czrB* gene is a component of the *czrAB* operon suggested to play a role in the transport of zinc across the cell membrane (Kuroda et al., 1999). The operon functions to maintain appropriate intracellular concentrations of this element, which is a prosthetic factor of many intracellular enzymes. The *czrB*, coding for a 36 kDa membrane spanning protein, was found to be homologous to the *czcD* gene, cobalt, zinc and the cadmium-resistant factor of *Bacillus subtilis* and *Alcaligenes eutrophus*. An association between truncation of CzrB in 49SCV and its phenotype is disputable, but possible, taking into account the fact that at higher concentrations, which may result from dysfunction of the regulatory CzrAB operon, zinc inhibits bacterial growth rate (Kuroda et al., 1999).

A nonsense mutation was detected in the NPW32_RS07065 locus encoding a truncated (34 aa/355 aa) nitric oxide synthase oxygenase (NOS) enzyme. Wang et al. suggested that the NOS gene can negatively regulate biofilm formation in SE. Disruption of the NOS gene resulted in an enhanced biofilm formation coupled with its weakened dispersal, as well as a slight retardation of bacterial growth and a decreased autolysis rate (Wang et al., 2022). Nitric oxide was also reported to increase bacterial resistance to antibiotics, which can be achieved both by the chemical modification of toxic compounds and the alleviation of the oxidative stress imposed by antibiotics (Gusarov et al., 2009). Kinkel et al. showed that NOS produced by SA, in concert with a NO·-metabolising flavohaemoprotein, regulates electron transfer by targeting haem-containing cytochrome oxidases under microaerobic conditions to maintain membrane bioenergetics (Kinkel et al., 2016). Following these literature reports, we can assume that disruption of the *nos* gene in 49SCV could have been related both to its strong biofilm production capability, but also to sensitivity to antibiotics, with the exception of gentamicin resistance. The 49SCV strain has been assumed to develop its gentamicin resistance due to genetically-based defects in electron transport. A supporting role of NOS dysfunction in this regard, however, cannot be excluded.

#### Single nucleotide polymorphisms (SNPs) identified in 49SCV

**Figure 6:**
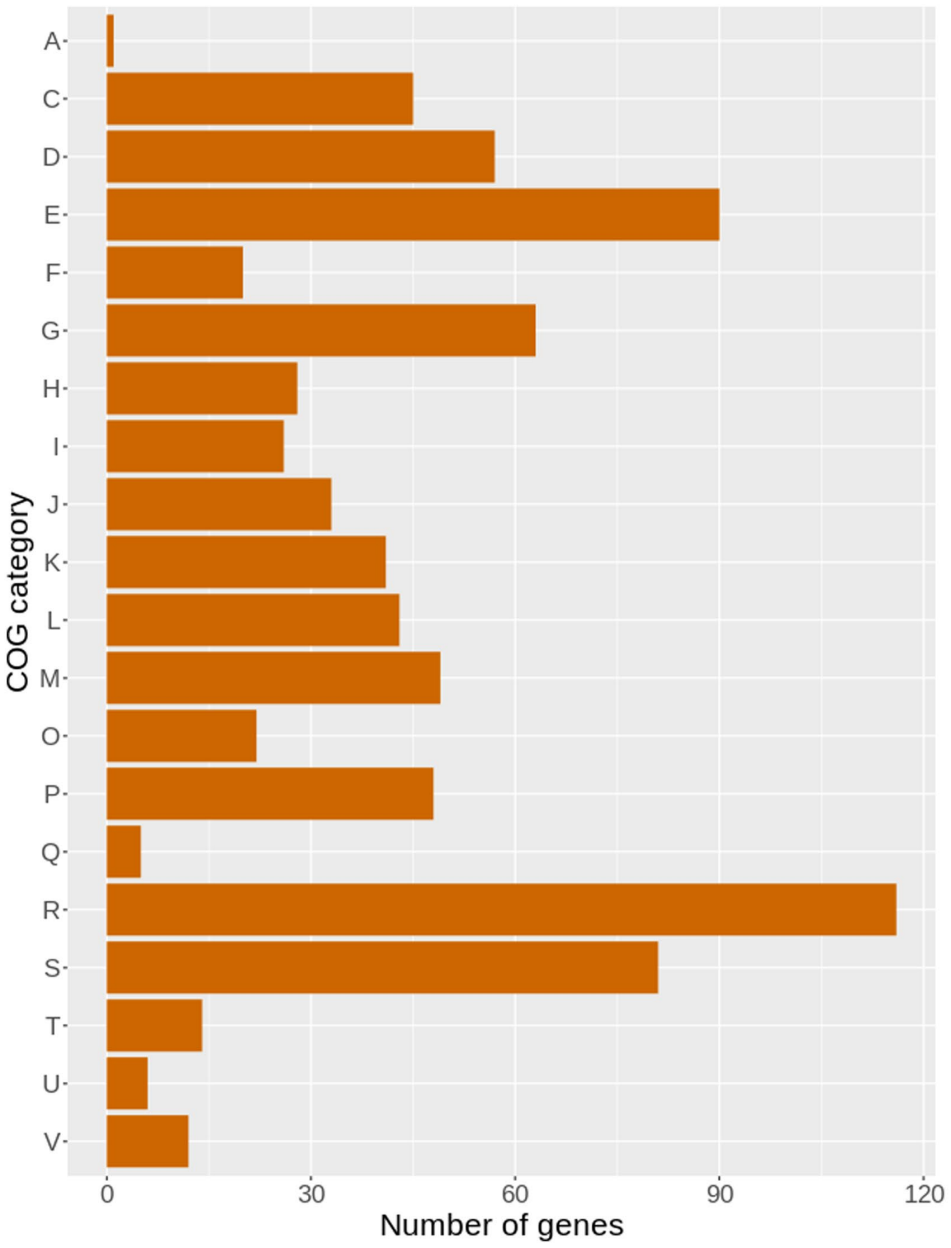
The bar plot illustrating the distribution of genes with single nucleotide polymorphisms (SNPs) in the genome of 49SCV, compared to the reference O47 genome, and clustered within different COG categories.

Point mutations have been evidenced to have a limited impact on cellular metabolism of SE SCV compared to the frameshift mutations (Liu et al., 2021). Using the reference O47 genome, the set of nonsynonymous single-nucleotide variations were identified in 49SCV. The set covered 713 genes, involved in a wide range of metabolic processes. Based on the amino acid sequence, COG categories were assigned to these genes. Mutated genes were distributed into 20 of the 26 functional categories. The overrepresented COG categories included: amino acid transport and metabolism (E), carbohydrate transport and metabolism (G), cell wall/membrane/envelope biogenesis (M), inorganic ion transport and metabolism (P), general function prediction (R), and unknown function (S) (Figure 6).

As the WT counterpart of the 49SCV strain was not cultured from the patient, we assume that a great number of SNPs identified against the O47 strain could be ascribed to the natural level of variability between the strains. In order to obtain a more complete picture and to find SNPs hypothesized to be directly responsible for the SCV phenotype formation, we analyzed the pangenome constructed from the genome of 49SCV and the five reference SE genomes. This analysis was conducted in the search of gene clusters for which the predicted amino acid sequences were specific only to 49SCV and remained constant in the other reference strains. We identified 178 variable gene clusters (Supplementary File 2), among which 22 had a unique sequence at the amino acid level in 49SCV in comparison to the reference strains (Table 2).

Substitutions identified as unique to the 49SCV genome, can be associated either with the loss or gain of gene function. Although metabolic analyses will be necessary to ascertain whether these genetic mutations reprogrammed the metabolism of the stable 49SCV strain, several hypotheses regarding the biological significance of identified SNPs in 49SCV can be corroborated by the results of phenotypic analysis of 49SCV published previously (Bogut et al., 2014).

Of note, SNPs were detected in the *menA*, *menH*, and *hemN*(*hemW*) genes whose products are involved in menaquinone and haem biosynthesis, respectively. A frameshift mutation in the HT of the *hemA* gene was also detected, which, as discussed earlier, could have been sufficient to develop an electron-transport-deficient SCV phenotype. Although it is conceivable that the above mentioned SNPs might have had their contribution to the phenotype transition, our previous study (Bogut et al., 2014) revealed that 49SCV did not demonstrate menadione auxotrophy. The potential role of SNP detected in the *hemN*, encoding for an oxygen-independent coproporphyrinogen-III oxidase, is also under speculation. Oxidative decarboxylation of coproporphyrinogen III to protoporphyrinogen IX mediated by HemN in *E. coli* can be catalyzed by the *hemF* gene product as well, which is active in the presence of oxygen (EcoCyc, 2020). The *E. coli hemN* mutant was found defective for the cytochrome b and cytochrome c synthesis only under anaerobic growth conditions (Tyson et al., 1997) which makes a compensatory role of HemF oxidase conceivable.

SNP detected in the gene encoding for the alternative sigma factor B (SigB; σ^B^) represents a genetic event whose contribution to 49SCV phenotype and its related characteristics could indicate an intact or even increased biological activity. SigB is an important transcription factor in staphylococci that is associated with responses to environmental stresses via redirection of transcriptional priorities (Tuchscherr et al., 2015). SigB activity has been described to influence the production of virulence factors, biofilm formation and intracellular persistence in SCVs (Mitchell et al., 2013). SigB may positively influence the appearance of SA SCVs and the production of biofilm upon aminoglycoside exposure (Mitchell et al., 2010). Tuchscherr et al. (2015) demonstrated that SigB enables SA to switch from the highly aggressive phenotype involved in acute infections to the SCV phenotype associated with a long-term intracellular persistence. In their study, Δ*sigB*-mutants failed to generate SCVs and were completely cleared by the host cells within a few days. SigB was also reported to silence the *agr* system which is involved in the enhanced inflammatory activity (Bischoff et al., 2001). The homology of the nucleotide sequence and the organization of the *sigB* operon in SE suggest a general function similar to that observed for SA (Knobloch et al., 2001). Several reports described a crucial role for σ^B^ in SE pathogenesis with the prominent example of biofilm formation and the σ^B^-dependent expression of PIA (Knobloch et al., 2001, 2004). Positive regulation of the biofilm formation in SE by σ^B^ was reported to rely on transcriptional activation of *icaADBC* and assumed to be mediated by a negative transcriptional control of IcaR (Conlon et al., 2002; Handke et al., 2007). Hence, in view of a prominent PIA-dependent biofilm production in 49SCV (Bogut et al., 2014), it can be speculated that the amino acid substitution in SigB either did not affect or even enhanced its functionality, e.g., through modulation of interactions with specific promoter or regulatory proteins.

As many as seven SNPs were identified in the 49SCV *clpP* gene which may indicate the loss or impairment of the biological activity of its product. The gene encodes a protease involved in the regulation of the toxin-antitoxin (TA) system. The toxin and antitoxin genes are coexpressed to form a TA complex. MazEF is an important example of the TA module in SA (Fu et al., 2007). MazE represents an antitoxin protein that binds to the toxin MazF, thereby preventing its activity. MazF has RNase activity, which targets selected mRNAs (including *sigB, hla, spa* mRNAs) in SA. The ATP-dependent protease pair, ClpPC, serves as the functional unit for the degradation of the MazE antitoxin, thereby ensuring MazF toxin release and activation. Overproduction of MazF toxin was reported to arrest bacterial growth and assumed to be involved in persistence (Magnuson, 2007; Donegan and Cheung, 2009; Donegan et al., 2010; Proctor et al., 2014). On the other hand, low levels of ATP and ClpP as well as the higher levels σ^B^ in electron transport SCVs suggest that the MazEF system may not contribute to persistence in electron transport SCVs (Proctor et al., 2014). The latter assumption is in line with our hypothesis on the genetically induced dysfunction of the ClpP protease and an intact SigB (discussed above) in the 49SCV electron-deficient strain. It is conceivable that the dysfunctional ClpP is not capable of the MazE antitoxin cleavage; hence, the MazF toxin probably cannot be released and exert its action on the *sigB* mRNA.

SNPs were detected in the genes encoding for the protein export systems SecA and SecA2. Since Sec proteins are involved in delivering proteins from the cytoplasm to the cell envelope or extracellular environment, their role is considered critical in terms of bacterial viability, physiology and pathogenesis. Proteins whose transport is mediated by the Sec pathway are synthesized as preproteins equipped with N-terminal signal peptides that are recognized by the Sec machinery. These peptides are cleaved during export across the inner membrane to produce mature proteins. SecA is a key component of the general protein secretion machinery. It participates in recruitment and delivery of suitable substrates to the SecYEG channel and functions as an ATPase motor to provide energy for the export. The main catalytic moiety of SecA is the ‘DEAD’ motor formed by the nucleotide binding domain and the intramolecular regulator of ATPase activity 2 domain. The ATP binding site of SecA is located at the interface of the two domains (Chaudhary et al., 2015). SecA2s is another transport-associated ATPase. It is a part of the accessory Sec (aSec) system that includes SecY2 (a paralogue of SecY) and accessory Sec proteins (Asps). Of note, the aSec system mediates the export of serine-rich repeat glycoproteins that function as adhesins (Chatzi et al., 2014; Jin et al., 2018; Braunstein et al., 2019; Ambroziak et al., 2021). In the study of Siboo et al. (2005), disruption of the *secA2* in SA resulted in the near complete loss of SraP adhesin expression on the cell surface. The loss of this platelet-binding adhesin expression has been linked to a reduced SA virulence in a model of endovascular infection (Siboo et al., 2005). Amino acid substitution in the SecA protein of 49SCV was found distant from the DEAD motor encoding region, near its C-terminal domain. However, the C-terminal domain along with the preprotein binding domain (PBD) contribute to the SecA substrate specificity (Chaudhary et al., 2015). Substitution in the SecA2 protein was detected within the ATP-binding site. The ATP-binding properties of the protein domains were reported to be essential for SecA-dependent translocation ATPase and protein translocation in *E. coli* (Mitchell and Oliver, 1993). Amino acid substitutions in the Walker A motif of SecA2 affected ATP binding *in vitro* in the study of *Mycobacterium tuberculosis* (Hou et al., 2008). We assume that SNPs in the genes representing the Sec system components might have led to the modulation of efficiency of the transport of Sec-dependent proteins making an association with a prolonged growth of 49SCV and chronicity of the PJI conceivable.

The two-component regulator system *vraSR* (vancomycin-resistance associated sensor/regulator) has been postulated to play several roles important for bacterial viability, virulence, and antibiotic resistance. It constitutes a positive regulator of the cell wall biosynthesis and is involved in the expression of beta-lactam and glycopeptide resistance in SA. Additionally, VraSR can regulate transcription of genes encoding stress-response proteins including proline/glycine-betaine (osmoprotectant) transporters (*proP* and *opuD*) (Kuroda et al., 2003; Cui et al., 2009). It the study of Dai et al. increase in the MICs of vancomycin was correlated with an increased *vraR* expression and a decreased expression of virulence genes (*hla*, *hlb*, and *coa*) and virulence-regulated genes (RNAIII, *agrA*, and *saeR*) suggesting that VraR might also regulate SA virulence (Dai et al., 2017). Interestingly, the *vraRS* genes were found among the down-regulated genes following a transcriptomic analysis of a stable SA SCV strain isolated from a patient with a PJI relapse. Downregulation of energy pathways and *vraRS* was considered in line with the emergence of the SCV phenotype and the switch from acute to chronic infection for isolates of clinical origin (Loss et al., 2019). The SNP-mediated modulation of the *vraR* gene function can also be assumed for the 49SCV strain which was additionally devoid of the functional ADI pathway and characterized by electron-deficiency due to hemin-auxotrophy.

## Conclusion

This study demonstrates novel genetic mechanisms that can be involved in staphylococcal phenotype switching. We propose that previously unreported indels in the HTs can constitute a background of this phenotype due to a resulting truncation of the corresponding proteins and their possible biological dysfunction. The strongest evidence for our hypothesis is a deletion in the poly(A) tract of the *hemA* gene, considered a possible trigger factor for phenotype switching and hemin auxotrophy in 49SCV. Streamline of the genetic content evidenced by the absence of the SCC element and a large ~12 kb deletion can represent a strategy associated with the development of the SCV phenotype and its adaptation to chronicity. Future studies based on transcriptomic and proteomic analyses are expected to shed more light on the actual association between genetic changes and the expression of mutated genes. They will be important to improve our understanding of molecular mechanisms driving phenotype switching and pathogenesis of PJIs in which staphylococcal SCVs are involved.

## Data availability statement

The datasets presented in this study can be found in online repositories. The names of the repository/repositories and accession number(s) can be found in the article/Supplementary material.

## Author contributions

AB: Conceptualization, Data curation, Formal analysis, Funding acquisition, Investigation, Methodology, Project administration, Resources, Supervision, Visualization, Writing – original draft, Writing – review & editing. PK: Data curation, Formal analysis, Investigation, Methodology, Software, Visualization, Writing – original draft, Writing – review & editing. MM: Conceptualization, Data curation, Formal analysis, Investigation, Methodology, Validation, Visualization, Writing – original draft, Writing – review & editing. PC: Formal analysis, Investigation, Methodology, Software, Validation, Writing – review & editing.
